# Did the COVID-19 Pandemic Affect Contrast Media-Induced Adverse Drug Reaction’s Reporting? A Pharmacovigilance Study in Southern Italy

**DOI:** 10.3390/jcm11175104

**Published:** 2022-08-30

**Authors:** Claudia Rossi, Rosanna Ruggiero, Liberata Sportiello, Ciro Pentella, Mario Gaio, Antonio Pinto, Concetta Rafaniello

**Affiliations:** 1 Department of Radiology, CTO Hospital, Azienda Ospedaliera dei Colli, 80131 Naples, Italy; 2 Department of Experimental Medicine, University of Campania “L. Vanvitelli”, 80138 Naples, Italy; 3 Campania Regional Centre for Pharmacovigilance and Pharmacoepidemiology, 80138 Naples, Italy

**Keywords:** contrast media, safety, spontaneous reporting, COVID-19

## Abstract

Medical imaging is required for a complete clinical evaluation to identify lung involvement or pulmonary embolism during SARS-CoV-2 infection or pulmonary and cardiovascular sequelae. Contrast media (CM) have undoubtedly been useful in clinical practice due to their ability to improve medical imaging in COVID-19 patients. Considering their important use, especially in hospitalized COVID-19 patients, and that increased use of a medical tool could also be associated with its deeper knowledge, we chose to explore if new information emerged regarding CM safety profiles. We analyzed all Individual Case Safety Reports (ICSRs) validated by Campania Pharmacovigilance Regional Centre from 1 January 2018 to 31 December 2021 and reported a CM (ATC code V08) as a suspected drug. We compared CM-related reporting between 2 years before (period 1) and 2 years during (period 2) the COVID-19 pandemic. From our analysis, it emerged that, during the COVID-19 pandemic, CM-related ADR reporting decreased, but a significant increase in reporting of serious cases emerged. Serious ADRs were mainly related to iodinated CM (V08A ATC) compared to magnetic resonance imaging CM (V08C ATC). Cutaneous and respiratory disorders were the most frequently reported in both periods. No new or unknown ADRs were reported in the overall study period.

## 1. Introduction

Since the outbreak of unknown pneumonia in Wuhan (China) at the end of 2019, the new coronavirus, identified as severe acute respiratory syndrome coronavirus 2 (SARS-CoV-2), has required global attention [1,2]. SARS-CoV-2 succeeds in entering the human cells through the spike protein present on its envelope surface, binding the angiotensin-converting enzyme 2 (ACE2) receptors and subsequentially using the serine protease TMPRSS2 for spike protein priming [3]. Once in the cell, its transcription and replication start exploiting the host cellular structures, causing an infection termed coronavirus disease 2019 (COVID-19) [1]. COVID-19 has posed an extraordinary threat to global public health [4]. The major impact on health systems was due to the increased hospitalization rates required for severely affected patients, especially in the first pandemic phases related to the more pathogenic SARS-CoV-2 variants (alpha and delta). According to the latest data, as of 23 May 2022, there have been 522,783,196 confirmed COVID-19 cases, including 6,276,210 deaths, reported to the WHO since the beginning of the pandemic [5]. Interstitial pneumonia represents the predominant clinical manifestation of mild and severe COVID-19 forms. Thromboembolic complications, including pulmonary embolism (PE) [6,7,8], are very frequent in COVID-19 patients [9,10,11]. Computed tomography (CT) has represented the reference standard for identifying lung involvement due to SARS-CoV-2 infection [12]. CT angiography is the key technique used for confirming pulmonary embolism [13]. In addition to respiratory complications, COVID-19 can also have implications on the cardiovascular system [14]. These include myocardial edema, fibrosis, and impaired right ventricle function, as revealed by cardiovascular magnetic resonance (CMR) [15,16]. Moreover, several long-term manifestations related to SARS-CoV-2 infection should not be excluded or underestimated. Long COVID-19 syndrome is characterized by several complications and sequelae, including pulmonary and cardiovascular ones, which require medical imaging for their clinical evaluation [17,18]. Therefore, contrast media (CM) have undoubtedly been clinically useful in the pandemic context due to their ability to improve medical imaging in COVID-19 patients [19]. Newer contrast agents, characterized by lower ionic concentrations and lower osmolarity, are better tolerated [20]. However, CM, like all medicines, is not risk-free [21], with possible negative effects, for example, on kidney function, especially in previously nephropathic patients when exposed to iodine-based agents [13]. CM-induced nephropathy has been the object of several studies that aimed to identify possible risk factors or biomarkers and evaluate procedures to prevent it [21]. Moreover, non-renal complications can also emerge following CM exposure, including anaphylactoid reactions (Krause et al., 2020). Post-marketing surveillance had an important role in identifying some CM-induced adverse drug reactions (ADRs), especially in some sub-populations with less evidence available due to their typical exclusion from clinical trials [19]. Considering the important use of CM for clinical imaging evaluations during the COVID-19 pandemic, especially in hospitalized patients, and that increased use of a medical tool could also be associated with its deeper knowledge, we wanted to explore if new information emerged regarding their safety profiles. For these reasons, we decided to investigate the effects of the COVID-19 pandemic on reporting suspected ADRs related to CM agents to the Italian National Pharmacovigilance Network (Rete Nazionale di Farmacovigilanza, RNF).

## 2. Materials and Methods

### 2.1. Data Source

For our study, we analyzed the individual case safety reports (ICSRs) related to CM and recorded them in the Campania Region (Southern Italy). Regional safety data were obtained from the RNF, the Italian Pharmacovigilance Database, coordinated by the Italian Medicines Agency (AIFA) since 2001. It allows for collecting Italian reports of suspected ADR and AEFI sent by physicians, other healthcare professionals and patients/citizens. ADR reporting is carried out through a standardized reporting form to describe details of the patient who experienced an adverse event (age, sex, medical history, etc.), the suspected ADR(s)/AEFI(s) (signs and symptoms or diagnosis, seriousness, outcome, etc.), the suspected and any concomitant drug(s)/vaccine(s) as well as previous or current patient medical conditions. In each Italian regional territory, Pharmacovigilance Regional Centers validate the information of each ICSR and perform the causality assessment for each adverse event–drug couple.

### 2.2. Study Design

We compared the CM-related reporting before and during the COVID-19 pandemic for our analysis. Therefore, we selected all ICSRs validated by the Campania Pharmacovigilance Regional Center from 1 January 2018 to 31 December 2021 and reported a contrast media (Anatomical Therapeutic Chemical classification code, ATC code V08) as a suspected drug. This reference period was chosen to compare the main features of ADRs’ reports entry into RNF during the 2 years before and during the COVID-19 pandemic (period 1: 1 January 2018 to 31 December 2019; period 2: 1 January 2020 to 31 December 2021).

### 2.3. Data Analysis

We performed a descriptive analysis of selected ICSRs, comparing and stratifying the two study periods by suspected contrast media, mean age, sex, and seriousness criteria according to the International Conference on Harmonization E2D guidelines principles (death; hospitalization or its prolongation; severe or permanent disability; life threat; congenital abnormalities/birth deficits; clinically relevant; not serious), outcome (favorable: completely resolved or improved; unfavorable: resolved with sequelae or unchanged), system organ class (SOC), High-Level Group Term (HLGT) MedDRA, source of the report, and causality assessment. We used MedDRA version 25.0. We evaluated the CM-related reporting trend stratifying by three-month/year.

Chi-squared analysis with Yates’ continuity correction or Fisher’s exact test, where appropriate, and *t*-test were employed to examine differences in the rate of the ADR’s report between the two periods. A 5% significance level was considered for analysis. Data were analyzed using the software SPSS version 21.

## 3. Results

From 1 January 2018 to 31 December 2021, a total of 144 ICSRs, describing 246 ADRs induced by CM, were sent to the Campania Pharmacovigilance Regional Center. All ICSRs were sent by a healthcare professional (Table 1).

**Figure 1:**
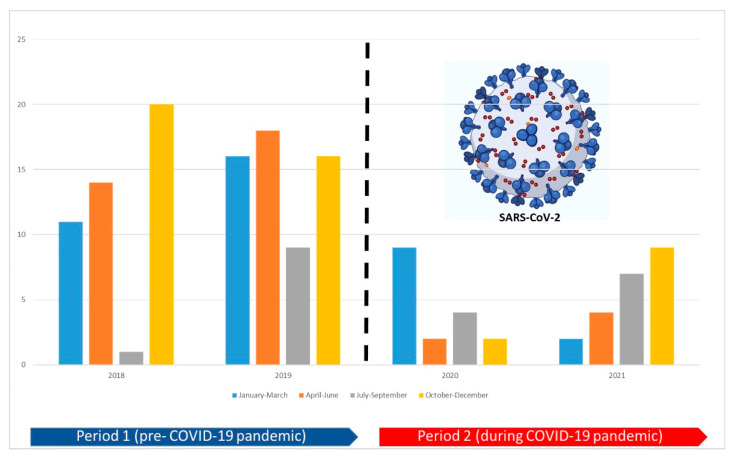
Before and during the COVID-19 pandemic, reporting trends stratified by three months/year.

As reported in Figure 1, we found a substantial decrease in CM-related reporting trends during the COVID-19 pandemic (105 ICSRS for period 1 vs. 39 ICSRs for period 2).

Overall, the mean age of patients who experienced ADRs was 55 years old (±16.1), and 53.5% were female (Table 1). These variable trends persist in both periods, even if the mean age was slightly higher in period 2, and the female sex is slightly less predominant in period 1. No significant differences in terms of age and sex between ICSRs sent to the RNF during periods 1 and 2 were found (Table 1). Considering the whole study period, the most commonly suspected drugs were iodinated X-ray CM (V08A ATC), reported in 84% of ICSRs. Among these, iopamidol (*n* = 63; 43.8%) and iomeprol (*n* = 32; 22.2%) were the most frequently reported. This distribution also persisted in both considered periods, before and during the COVID-19 emergency. Magnetic resonance imaging CM (ATC V08C), in particular gadoteric acid and gadoteridol, were the other CM class reported as suspected drugs in the retrieved ICSRs. Among CM belonging to V08C ATC, comparing the two periods, the most frequently reported were gadoteric acid in period 1 (*n* = 9; 8.6%) and gadoteridol in period 2 (*n* = 3; 7.7%). Moreover, we found 2 ICSRs sent during period 1 reporting other suspect drugs, in particular chlorhexidine and mepivacaine (Table 1). CM was mainly used for the CAT scan (data not shown). Regarding the outcome of ADRs’ reports, overall, 86.8% of collected ICSRs had a favorable outcome. Comparing the two periods, we found an increase in the number of ICSRs with favorable outcomes (82.9% in period 1 vs. 97.4% in period 2). This difference was statistically significant (*p* = 0.04) (Table 1). Overall, a seriousness degree was available for 139 reports: 75.7% of cases were classified as not serious, while only about 21% were classified as serious. Stratifying results by reference periods, the percentage of serious ICSRs increased in period 2 (16.2% in period 1 vs. 33.3% in period 2) (Table 1). Additionally, this difference was statistically significant (*p* = 0.04) (Table 1). Since each ICSR can report more than 1 ADR, overall, 77 serious ADRs were described in 30 ICSRs sent to Campania Regional Centre (17 in period 1 vs. 13 in period 2) (Table 2). Among those 77 serious ADRs, 90% were related to iodinated CM (V08A ATC), while the remaining 10% were related to magnetic resonance imaging CM (V08C ATC) (Table 2).

Comparing the two-reference period, this distribution, with the major involvement of V08C in the occurrence of serious ADRs, was confirmed. Iopamidol (37.7%) and iomeprol (23.4%) persisted as the more frequently iodinated CM involved in serious cases (V08A ATC). Regarding V08C CM, we found a few serious cases related to gadobenic (*n* = 5) and gadoteric (*n* = 1) acids reported only in period 2, while the only serious ICSR related to gadoteridol was reported in period 1. As reported in Table 2, no statistically significant difference emerged.

**Figure 2:**
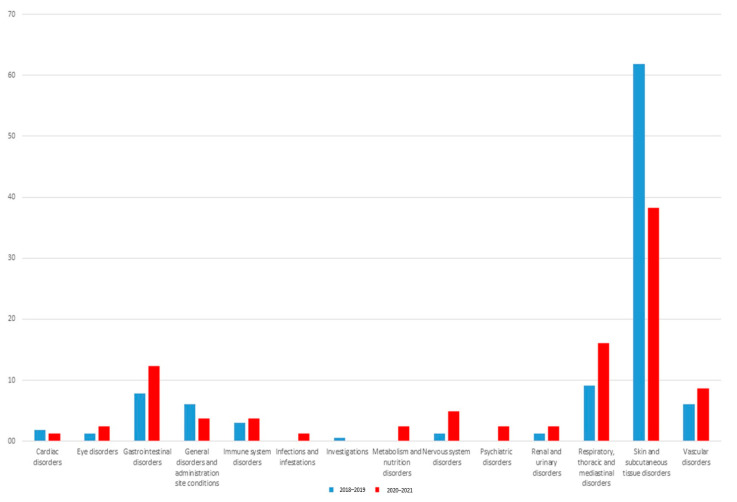
ADR distributions by system organ class in ICSRs reporting contrast media as suspected drugs and collected in the Campania Region. Comparison between pre-COVID-19 pandemic (period 1 = 2018–2019; blue color) and during the COVID-19 pandemic (period 2 = 2020–2021; red color).

The distribution of ADRs by SOC and HLGTs in two reference periods is shown in Figure 2 and Table 3, respectively.

“Skin and subcutaneous tissue disorders” (61.8% in period 1 vs. 38.3% in period 2) and “respiratory, thoracic and mediastinal disorders” (9.1% in period 1 vs. 16% in period 2) were the first two SOCs more frequently reported in both periods. Moreover, gastrointestinal (7.9% in period 1 vs. 12.3% in period 2) and general disorders (6.1% in period 1 vs. 3.7% in period 2) were commonly reported, even if less frequently in period 2, as per the general trend. Vascular disorders represented 6.1% of reported ADRs in period 1 and 8.6% of those reported in period 2. Otherwise, we found a more frequent reporting of ADRs belonging to “nervous system disorders” SOC in period 2 (1.2% vs. 4.9%). Regarding CM renal toxicity, ADRs belonging to “renal and urinary disorders” SOC were reported only in a few ICSRs in both periods, representing 1.2% vs. 2.5% of reported ADRs in the two reference periods, respectively (Figure 2). Comparing two reference periods, statistically significant differences emerged for “epidermal and dermal conditions” and “oral soft tissue conditions” HLGTs (Table 3). CM belonging to V08A ATC was always the most involved category in reporting ADRs per all SOCs (data not shown).

**Figure 3:**
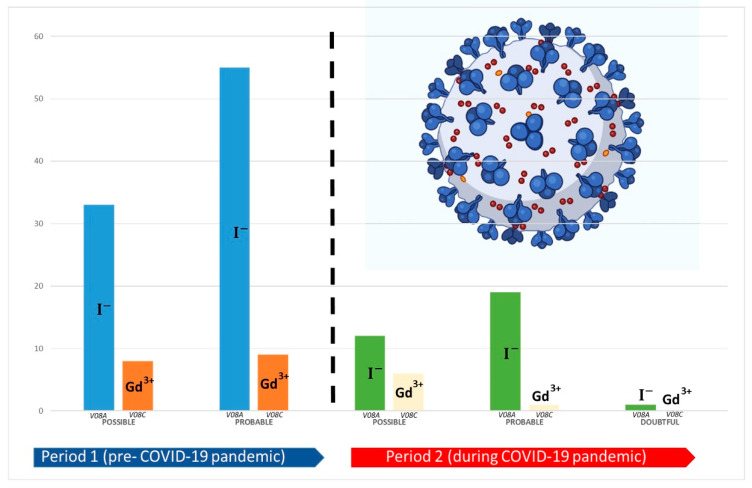
Distribution by causality assessment of ICSRs reporting contrast media as suspected drugs and collected in the Campania Region from 1 January 2018 to 31 December 2021. Comparison between pre-COVID-19 pandemic (period 1 = 2018–2019) and during the COVID-19 pandemic (period 2 = 2020–2021). Contrast media are categorized for III levels of ATC classes. V08A: X-ray contrast media, iodinated; V08C: magnetic resonance imaging contrast media.

Considering serious ADRs, allergic or anaphylactic reactions were the more frequent ones reported during period 1, which required/prolonged patient hospitalization or represented life-threatening. These ADRs included mild manifestations such as allergic purpura or gum and mouth swelling and important systemic adverse events like anaphylactic shock. In the same period, flushing, erythematous skin rash, and hives are some examples of not serious ADRs that were more frequently reported. In particular, iopamidol was involved in 3 cases of anaphylactoid reaction reported in period 1 and 2 cases of reversible ischaemic neurological deficit reported in period 2. The remaining serious ADR reports were mainly related to hypotension or hypertensive crisis, cardiac rhythm disorders (including tachycardia and bradycardia and a case of cardiac arrest). Moreover, only one case reported in period 2 described toxic renal events that occurred in a 60-year-old female treated with iopromide for abdominal CAT, who experienced dependent edema, hyperkalaemia, acidosis metabolic, oliguria and renal failure, which required patient hospitalization. Finally, following Naranjo’s algorithm computation, the causality assessment was shown to be probable (61%) for the majority of period 1 ICSRs, while the remaining 39% were evaluated as possible. Regarding period 2, the causality assessment resulted in possible, probable or doubtful assessments in 46.2%, 51.3% and 2.6% of cases, respectively (Figure 3). Distributions of cases by causality assessment in the two reference periods are reported in Figure 3, in which CM is categorized for III levels of ATC classes (V08A and V08C).

## 4. Discussion

The COVID-19 pandemic has had several impacts on society, influencing various aspects related to drugs, such as pharmacovigilance and spontaneous reporting. Safety assessment and issues related to anti-COVID-19 vaccines have turned the spotlight on pharmacovigilance systems and mechanisms. Due to the media and civil attention focused on COVID-19 vaccines, the reporting by citizens has considerably increased during the pandemic. Nevertheless, from our analysis, the reporting of ADR induced by CM has not been the object of interest for patients and citizens. In fact, healthcare professionals sent all CM-related ICSRs in our study period. Generally, the COVID-19 pandemic has negatively influenced CM-related ADR reporting. Several reasons can justify this result. Firstly, the observed substantial decrease in the CM-related reporting trend is in line with reporting national trends registered during the COVID-19 pandemic. According to the 2020 Vaccine Report published by the Italian Medicines Agency, the reporting rate for all vaccines (excluding COVID-19 vaccines) showed a sharp decline compared with recent years [22]. Primary sources, limited access, and attention focused on global emergencies can certainly be involved in the generally reduced reporting, including CM-related ones.

Generally, doctors, followed by nurses and other health workers, represent the primary sources of spontaneous reports, especially in South Italy [23,24,25,26,27,28,29,30,31]. Several initiatives have been implemented, and others are still necessary to implement a pharmacovigilance culture among all healthcare professionals and the general population [32,33,34,35,36,37]. The pressure to which healthcare personnel have been subjected during the COVID-19 emergency is well known. This was especially noted for those working in departments/wards dedicated to treating COVID-19 patients, for which medical imaging using CM was required for a complete clinical evaluation. Moreover, radiology departments were widely involved in the pandemic context since the key role of clinical imaging in diagnosing and managing COVID-19 patients [38]. In light of this, the observed reduction in reporting trends can be easily understood and expected. Moreover, the public health emergency has monopolized attention and energy in all fields, including pharmaco- and vaccine vigilance. In fact, even if these activities were implemented during the pandemic, they were strongly focused on vaccines and drugs repositioned against SARS-CoV-2 infection [39]. Finally, the decreased trend is likely attributed to lower patient access to non-emergency or essential health services, including diagnostic investigation [40]. Patients’ fear and the health pressure on hospitals and medical centers, as well as the limitations imposed by lockdowns and quarantines, have certainly been involved in this last aspect. However, if their wide use for clinical evaluation of hospitalized COVID-19 patients is considered, the reduced CM-related reporting during the pandemic should be in contrast with what is expected. Even if the COVID-19 pandemic was characterized by a decrease in elective diagnostic imaging procedures and clinical imaging, radiology departments played an essential part in detecting and following up on patients with COVID-19 during the pandemic [41,42]. Diagnostic procedures, such as CT, resulted in the fundamental disease course and severity assessment tools. Their usefulness in clinical management orienting was also confirmed during the COVID-19 pandemic. In fact, imaging techniques are included as part of the integrated approach necessary for diagnosis, monitoring and identification of the most appropriate therapy in COVID-19 patients, especially when hospitalized [41,42]. Compared to previous viral pandemics, imaging modalities played an essential role in the respiratory tract, cardiovascular, neurologic, and gastrointestinal evaluation of COVID-19 patients [41]. However, according to national data published by AIFA, a decrease in consumption and expenditure of CM has been registered in 2020. This decrease was probably due to a lower number of diagnostic tests carried out for chronic diseases during the first year of the COVID-19 pandemic [43]. Data related to Italian consumption and expenditure in 2021 are still not available. According to our results, iodinated CM, particularly iomeprol and iopamidol, were the most frequently involved in spontaneous reporting. Especially during period 2, they were mainly used for CAT scans. Instead, no ICSR reported angiograph coronary as their therapeutic indication. However, CT coronary angiography use increased during the COVID-19 pandemic since it was associated with a significantly reduced length of hospitalization both pre-COVID-19 and post-COVID-19 lockdown [44]. The strong effort of health care professionals engaged on the COVID-19 front can also be related to the increased reporting of serious ADRs emerging in period 2, with statistical significance. To emphasize this aspect, reporting attention was limited to the most severe adverse events. However, any new adverse events emerged during the two periods. Considering the event’s notoriety, all the ADRs reported in the overall study period were known and reported in the Summaries of Products Characteristics (SmPCs). In line with Sessa et al.’s results (Sessa et al., 2015a), our results also revealed that skin and respiratory disorders continued to be the more frequently reported ADRs in patients who received CM. As expected, the main reported ADRs were angioedema, urticaria and epidermal phenomena. Even if decreased in our study period, spontaneous reporting confirmed general toxicity more frequently related to iodinated CM compared to gadolinium-based CM. In fact, from our results, iodinated CM was more frequently involved in a major number of ICSRs, and serious ADRs, resulting in a more probable relationship with drug-adverse events according to the applied Naranjo algorithm. In the last years, attention has been given to CM nephrotoxicity among organ-specific adverse reactions, probably caused by induced endothelial dysfunction and renal cell apoptosis [21,45]. However, in our period study, renal disorders did not emerge as frequently, and only a few cases reported nephropathies or renal disorders. These were all related to iodine-based agents. In particular, a case of renal failure with oliguria associated with acidosis metabolic and hyperkaliemia, requiring hospitalization but favorably evolving, was reported in period 2. Considering CM-related organ-specific toxicity, cardiovascular is another important aspect that emerged, together with anaphylactic shock, among the most frequently reported fatal CM-related ADRs in the Campania Region [46]. According to our results, cardiac arrhythmias, including tachycardia and cardiac arrest, were other rarely reported ADRs that need close monitoring since they represent life-threatening for patients. Malignant arrhythmogenesis is not an uncommon complication of some imaging techniques, such as invasive coronary angiography [47]. Often, the main cause can be related to technical procedural issues. Following the trigger of such arrhythmic events can be the CM administration since iodine-based effects are influenced not only by their osmolarity but also by their sodium concentration and calcium-binding properties. It is well known that administering CM makes patients more susceptible to ventricular fibrillation or sustained ventricular [47]. Finally, neurotoxicity represents another CM-related organ-specific issue that recently emerged. A recent safety review of EMA confirmed in 2018 the possible accumulation of small amounts of gadolinium in the cerebral tissues following the use of linear gadolinium-based CM more than macrocyclic ones. Since the long-term risks associated with this phenomenon were unknown, the EMA was suspended in the EU gadolinium containing linear CM with the exception of gadoxetic acid and gadobenic acid, available exclusively for use in hepatic scans [48]. In line with these use restrictions, only a few cases related to these two CM agents emerged from our results. No cases of long-term nervous adverse events were reported.

In light of our results, the adopted measures against COVID-19 are likely to have an important impact on spontaneous reporting related to other medicine not specifically indicated to COVID-19 treatment, such as CM. Whilst our results could be reassuring regarding the safety profile of CM as drugs well-known and long been used in clinical practice, on the other hand, the pandemic was likely to be a missed opportunity to deepen the safety of drugs so important for the evaluation of patients and the course of COVID-19 and many other diseases.

## 5. Strengths and Limitations

Our study presents the well-known limitations related to the spontaneous reporting system, including possible under-reporting and inaccurate, incomplete or lack of clinical data [49,50]. Unavailable/not reported data can have an important impact on the results of pharmacovigilance studies, such as ours. Moreover, since we have decided to perform our pharmacovigilance analysis during 4 years of observation in one single Italian region, we have extracted a very limited number of reports, which should not be considered representative of the other Italian regions. Despite these limitations, we present a comprehensive evaluation of safety data related to CM in the Campania region during the COVID-19 pandemic and compare them with those emerging from the previous two years using the Italian spontaneous reporting system. Although the spontaneous reporting system has an intrinsic limitation, it is largely accepted that it represents a simple and inexpensive tool to detect rare and serious ADRs that have not emerged during premarketing clinical phases. Furthermore, this method allows for generating safety hypotheses on medicines that shall be confirmed or refuted by other ad hoc studies.

## 6. Conclusions

During the COVID-19 pandemic, the imaging techniques with CM achieved a better clinical evaluation of patients affected with COVID-19, especially if hospitalized, allowing an integrated approach to diagnosing, monitoring, and managing SARS-CoV-2 infection. Despite the wide use of CM during the COVID-19 pandemic, there was a decrease in ADR reporting. In our opinion, reasons for this are undoubtedly various, including the strong effort of health care professionals engaged on the COVID-19 front, with less time to spend in spontaneous ADR reporting and attention focused on the global emergency. Pharmacovigilance activities were more focused on vaccines or repurposed drugs used for COVID-19 treatment. Despite those effects on ADRs’ reporting, there is no doubt about the usefulness of the CM in imaging for a greater assessment of acute and chronic complications related to COVID, reducing the length of hospitalization. Although the pandemic was likely to be a missed opportunity to deepen CM safety, the continuous monitoring of CM safety and the full implementation of pharmacovigilance activities will play a key role in achieving optimal clinical imaging.

## Figures and Tables

**Table 1:** Main features of 144 ICSRs related to contrast media in the Campania region comparing the 2018–2019 (period 1) and 2020–2021 (period 2).

Variables	Levels	Total ICSR *n* = 144	Contrast Media ICSRs During Period 1 *n* = 105 *n* (%)	Contrast Media ICSRs During Period 2 *n* = 39 *n* (%)	*p*-Value (<0.05)
Age	Mean age (SD)	55 (±16.1)	54 (±16.9)	57 (±13.4)	0.26
Sex	Female n (%)	77 (53.5)	52 (49.5)	25 (64.1)	0.17
	Male n (%)	65 (45.1)	51 (48.6)	14 (35.9)	0.24
	Not Reported n (%)	2 (1.4)	2 (1.9)	-	-
Seriousness of ADRs’ reports	Serious n (%)	30 (20.8)	17 (16.2)	13 (33.3)	0.04
	Not Serious n (%)	109 (75.7)	83 (79.0)	26 (66.7)	0.18
	Not available	5 (3.5)	5 (4.8)	-	-
Outcome of ADRs’ reports	Favorable n (%)	125 (86.8)	87 (82.9)	38 (97.4)	0.04
	Unfavorable n (%)	1 (0.7)	1 (0.9)	-	-
	Not Available n (%)	18 (12.5)	17 (16.2)	1 (2.6)	0.05
Source of ADRs’ reports	Healthcare professional n (%)	144 (100)	105 (100)	39 (100)	-
Suspected drug					
V08A	Iomeprol n (%)	32 (22.2)	23 (21.9)	9 (23.1)	0.94
	Iopamidol n (%)	63 (43.8)	46 (43.8)	17 (43.6)	0.86
	Iopromide n (%)	11 (7.6)	5 (4.8)	6 (15.4)	0.07
	Ioexol n (%)	1 (0.7)	1 (0.9)	-	-
	Iodixanol n (%)	7 (4.9)	7 (6.7)	-	-
	Iobitridol n (%)	6 (4.2)	6 (5.7)	-	-
	TOTAL	121 (84.0)	88 (84.8)	32 (82.1)	0.99
V08C	Gadoteric acid n (%)	11 (7.6)	9 (8.6)	2 (5.1)	0.73
	Gadoteridol n (%)	9 (6.3)	6 (5.7)	3 (7.7)	0.96
	Gadobutrol n (%)	1 (0.7)	1 (0.9)	-	-
	Gadoxetic acid n (%)	1 (0.7)	1 (0.9)	-	-
	Gadobenic acid n (%)	2 (1.3)	-	2 (5.1)	-
	TOTAL	24 (16.0)	17 (15.2)	7 (17.9)	0.99
Other suspect drugs					
	Clorexidine n (%)	1 (0.7)	1 (0.9)	-	-
	Mepivacaine n (%)	1 (0.7)	1 (0.9)	-	-

V08A: X-ray contrast media, iodinated; V08C: magnetic resonance imaging contrast media.

**Table 2:** Distribution of serious ADRs associated with individual contrast-media categorized for 3rd level ATC.

	Number of Serious Suspected ADR in Total Contrast Media’s Reports *n* = 77	Number of Suspected Serious ADRs in Contrast Media’s Reports During Period 1 *n* = 41	Number of Suspected Serious ADRs in Contrast Media’s Reports During Period 2 *n* = 36	*p*-Value
V08A	70 (90)	40 (97.5)	30 (83.3)	0.07
Iomeprol	18 (23.4)	11 (26.8)	7 (19.4)	0.62
Iopamidol	29 (37.7)	13 (31.7)	16 (44.4)	0.36
Iopromide	11 (14.3)	4 (9.8)	7 (19.4)	0.37
Ioexol	2 (2.6)	2 (4.9)	0	0
Iodixanol	6 (7.8)	6 (14.6)	0	0
Iobitridol	4 (5.2)	4 (9.8)	0	0
V08C	7 (10)	1 (2.4)	6 (16.7)	0.07
Gadobenic acid	5 (6.5)	0	5 (13.9)	0
Gadoteric acid	1 (1.3)	0	1 (2.8)	0
Gadoteridol	1 (1.3)	1 (2.4)	0	0

V08A: X-ray contrast media, iodinated; V08C: magnetic resonance imaging contrast media. The total number of ADRs reported for each contrast media exceeds the total number of ICSRs since a single report might include more than one suspected ADR. Database from the Campania Region, Southern Italy.

**Table 3:** ADR distributions by high-level group terms (HLGTs) in ICSRs reporting contrast media as suspected drugs and collected in the Campania Region. Comparison between pre-COVID-19 pandemic (period 1: 2018–2019) and during the COVID-19 pandemic (period 2: 2020–2021).

	Period 1		Period 2		*p*-Value
High-Level Group Terms	N	%	N	%	*p*
Acid-base disorders	-	-	1	1.2	-
Allergic conditions	5	3.0	3	3.7	0.8
Angioedema and urticaria	27	16.4	10	12.3	0.4
Arteriosclerosis, stenosis, vascular insufficiency and necrosis	-	-	2	2.5	-
Body temperature conditions	1	0.6	-	-	-
Bronchial disorders (excl neoplasms)	1	0.6	1	1.2	-
Cardiac and vascular investigations (excluding enzyme tests)	-	-	1	1.2	-
Cardiac arrhythmias	3	1.8	1	1.2	0.7
Central nervous system vascular disorders	-	-	2	2.5	-
Decreased and nonspecific blood pressure disorders and shock	4	2.4	1	1.2	0.5
Deliria (including confusion)	-	-	1	1.2	-
Dental and gingival conditions	1	0.6	-	-	-
Electrolyte and fluid balance conditions	-	-	1	1.2	-
Epidermal and dermal conditions	73	44.2	18	22.2	0.0007
Eye disorders NEC	-	-	1	1.2	-
Gastrointestinal haemorrhages NEC	1	0.6	-	-	-
Gastrointestinal motility and defaecation conditions	-	-	1	1.2	-
Gastrointestinal signs and symptoms	10	6.1	5	6.2	0.9
General system disorders NEC	9	5.5	3	3.7	0.5
Infections—pathogen unspecified	1	0.6	-	-	-
Lower respiratory tract disorders (excluding obstruction and infection)	-	-	8	9.9	-
Nephropathies	2	1.2	-	-	-
Neurological disorders NEC	2	1.2	1	1.2	0.9
Neuromuscular disorders	-	-	1	1.2	-
Ocular infections, irritations and inflammations	2	1.2	1	1.2	0.9
Oral soft tissue conditions	1	0.6	4	4.9	0.02
Renal disorders (excluding nephropathies)	-	-	2	2.5	-
Respiratory disorders NEC	7	4.2	3	3.7	0.8
Respiratory tract signs and symptoms	6	3.6	-	-	-
Skin appendage conditions	1	0.6	3	3.7	0.07
Skin vascular abnormalities	1	0.6	-	-	-
Sleep disorders and disturbances	-	-	1	1.2	-
Upper respiratory tract disorders (excluding infections)	1	0.6	1	1.2	0.6
Vascular disorders NEC	6	3.6	3	3.7	0.9
Vascular hypertensive disorders	-	-	1	1.2	-

## Data Availability

Data supporting reported results can be found in the National Pharmacovigilance Network, but their availability is limited to Regional Pharmacovigilance Centers and the Italian Medicine Agency.
